# A Genome‐Wide Screening of Novel Immunogenic TrLSDV103 Protein of Lumpy Skin Disease Virus and Its Application for DIVA

**DOI:** 10.1096/fj.202500425R

**Published:** 2025-05-28

**Authors:** Xinwei Yuan, Junhua Dong, Zhijie Xiang, Qian Zhang, Pan Tao, Aizhen Guo

**Affiliations:** ^1^ National Key Laboratory of Agricultural Microbiology, College of Veterinary Medicine, Hubei Hongshan Laboratory Huazhong Agricultural University Wuhan China; ^2^ The Cooperative Innovation Center for Sustainable Pig Production Huazhong Agricultural University Wuhan China; ^3^ Hubei International Scientific and Technological Cooperation Base of Veterinary Epidemiology Wuhan China

**Keywords:** differentiating naturally infected from vaccinated animals, indirect ELISA, LSDV103 protein, lumpy skin disease virus, VirScan

## Abstract

Lumpy skin disease (LSD) is an infectious cattle disease caused by the lumpy skin disease virus (LSDV), posing a serious threat to the livestock industry. This study aimed to identify specific immunogenic targets for differential diagnosis and potential vaccine development. Using a phage display library covering the entire LSDV proteome, we screened sera from naturally LSDV‐infected cattle and those vaccinated with the live attenuated goatpox virus vaccine (GTPV AV41) to identify differential antibody‐binding viral peptides, leading to the identification of peptides within the LSDV103 protein. A truncated recombinant LSDV103 protein (TrLSDV103) was expressed and showed strong reactivity with sera from LSDV‐infected cattle, significantly higher than that with sera from GTPV‐vaccinated cattle. An indirect enzyme‐linked immunosorbent assay (iELISA) based on the TrLSDV103 protein was developed, with a cutoff value of 0.361, demonstrating the diagnostic specificity of 100% (95% CI: 90.11–100) and the diagnostic sensitivity of 86.67% (95% CI: 70.32–94.69). The lowest detection limit for positive serum was 1:6400, with no cross‐reactivity with five other bovine pathogens. Among 210 serum samples tested, 11 were suspected of LSDV infection. Additionally, TrLSDV103 protein immunization in BALB/c mice induced strong humoral and cellular immune responses, including significantly elevated IgG levels and antibody titers up to 1:204800. Cytokine detection assays showed a significant increase in IFN‐γ and IL‐1β levels. Flow cytometry analysis further revealed a marked increase in CD3^+^CD4^+^ T cells. In conclusion, TrLSDV103 is a highly immunogenic protein with strong diagnostic specificity, showing great potential as a differential diagnostic antigen and vaccine candidate against LSDV.

## Graphical abstract

Using the VirScan platform covering 4522 LSDV protein sequences, sera from 20 LSDV‐infected and 20 GTPV‐vaccinated cattle were used to screen phage libraries. Sequencing and bioinformatics analyses revealed peptides covering the TrLSDV103 protein. The candidate antigen was subsequently expressed, confirmed for its DIVA capability, and used to develop an iELISA. Furthermore, immunization of BALB/c mice demonstrated its strong immunogenicity, highlighting its potential as both a differential diagnostic antigen and a promising vaccine candidate against LSDV.

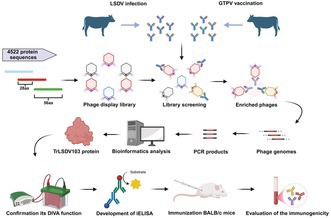

## 1 Introduction

Lumpy skin disease (LSD) is an infectious disease caused by the lumpy skin disease virus (LSDV), posing a serious threat to cattle health and the sustainable development of the livestock industry. Clinical signs in affected cattle include fever, enlarged lymph nodes, and nodular lesions on the skin and mucous membranes. These lesions were often complicated by secondary bacterial infections, leading to reduced productivity and economic losses [1, 2, 3]. LSD is classified as a notifiable disease by the World Organization for Animal Health (WOAH) [4]. LSDV exhibits high host specificity, primarily affecting cattle; although occasional infections have been reported in giraffes and antelopes, no human infections have been documented to date [5, 6, 7, 8, 9].

LSD originated in Africa and subsequently spread to Europe. In recent years, widespread outbreaks were reported across Asia, with incidence rates ranging from 5%–45%, and average mortality of approximately 5% [10]. As of the end of 2024, a total of 87 countries and regions had officially reported LSD outbreaks to WOAH (https://wahis.woah.org/#/home). LSD was first detected in China in 2019, initially in the Ili region of Xinjiang, before spreading to multiple provinces [11]. In 2022, China reclassified LSD as a Category II animal infectious disease.

LSDV belongs to the *Poxviridae* family and *Capripoxvirus* genus. It is a double‐stranded DNA, approximately 151 kb in length, encoding 156 putative genes with a GC content of 27% [12, 13]. The virus has an envelope structure and measures approximately 294 ± 20 nm in length and 262 ± 22 nm in width. It has only one serotype, exhibits no hemagglutination activity, and replicates in the cytoplasm [14, 15, 16].

China currently employs a fivefold goat dose of the live attenuated goatpox virus vaccine (GTPV AV41) for cattle vaccination, which provides good protection. However, due to the high antigenic similarity of up to 97% between LSDV, sheep pox virus (SPPV), and goatpox virus (GTPV), there is an urgent need for serological diagnostic methods capable of distinguishing between antibodies resulting from natural LSDV infection and those resulting from vaccination with the live attenuated goatpox vaccine [17, 18, 19].

Commercial diagnostic kits currently available are unable to distinguish antibodies generated by natural LSDV infection from those induced by GTPV vaccination. Previously, we developed an artificial protein called rLSDV‐gap by concatenating amino acid differences identified by genomic comparisons between LSDV and GTPV for differential diagnosis [20]. However, rLSDV‐gap is not a natural antigenic entity, and the artificially designed differences may not necessarily correspond to B‐cell epitopes capable of eliciting neutralizing antibodies, potentially limiting its ability to induce protective immunity against LSDV.

As LSDV‐infected and GTPV‐vaccinated cattle may induce different antibody responses, this indicates the existence of specific immunogenic peptide markers. In this study, we employed VirScan technology to identify LSDV‐specific peptide markers. VirScan is a high‐throughput approach that integrates phage display, immunoprecipitation, and large‐scale DNA sequencing, allowing comprehensive profiling of antiviral antibody responses and identification of immunodominant peptides [21, 22]. We constructed a phage display library presenting overlapping peptides spanning the entire LSDV proteome and performed VirScan screening using sera from LSDV‐infected and GTPV‐vaccinated cattle to identify LSDV‐specific peptide markers. Peptides corresponding to amino acids 1–135 of the LSDV103 protein were enriched in LSDV‐infected sera but not in GTPV‐vaccinated sera. Therefore, a truncated form of LSDV103 (amino acids 1–135, termed TrLSDV103) was expressed in *E. coli* BL21(DE3). TrLSDV103 exhibited differential diagnostic potential and was used to establish a DIVA method, which demonstrated high diagnostic sensitivity and specificity. In addition, the potential of TrLSDV103 as a candidate subunit vaccine was also evaluated. This strategy is expected to provide accurate technical support for the control, eradication, and purification of LSD in China's cattle industry.

## 2 Materials and Methods

### 2.1 Virus Strains

The study analyzed the nucleotide sequences of two viral strains: the lumpy skin disease virus strain China/GD01/2020 (GenBank accession no. MW355944.1) and the goatpox virus AV41 strain (GenBank accession no. MH381810.1).

### 2.2 Serum Samples

Phage immunoprecipitation was performed using 20 positive sera from cattle naturally infected with LSDV and 20 positive sera from cattle vaccinated with GTPV. Thirty‐five reference serum samples were collected from cattle 60 days post‐vaccination with a fivefold goat dose of the live attenuated goatpox vaccine, administered via intradermal injection. These samples were confirmed positive by both the virus neutralization test (VNT) and the ID Screen Capripox Double Antigen Multi‐species test kit (ID‐Vet, Grabels, France). These vaccinated sera were used to determine the cutoff value and to evaluate the diagnostic specificity of TrLSDV103 based iELISA.

Additionally, 30 reference serum samples were collected from cattle in China that were clinically and laboratory confirmed to be infected with LSDV. Serum antibodies against LSDV were confirmed positive by both VNT and ID Screen Capripox Double Antigen Multi‐species test kit (ID‐Vet, Grabels, France). These infected sera were used to assess the diagnostic sensitivity of TrLSDV103‐based iELISA.

Furthermore, 210 clinical serum samples with relevant backgrounds of naturally LSDV‐infected or GTPV‐vaccinated, or negative were examined to evaluate the clinical applicability of the TrLSDV103‐based iELISA. All serum samples were stored at −20°C until use.

### 2.3 Phage Immunoprecipitation

Phage immunoprecipitation was performed as previously described [23]. Briefly, 1.5 mL microcentrifuge tubes were blocked with 3% bovine serum albumin (BSA) at 4°C for 12–16 h. Cattle sera containing 2 μg of IgG and 2 × 10^8^ pfu phages were mixed in a 1.5‐mL microcentrifuge tube. After incubating at 4°C for 18 h with rotation, 40 μL of protein A/G magnetic beads (Thermo Fisher Scientific) were added and incubated for an additional 4 h at 4°C. The mixture was centrifuged at 900 *g* for 1 min at 25°C and placed on a magnetic stand to remove the supernatant. The magnetic beads were then resuspended in 600 μL of IP wash buffer (50 mM Tris–HCl, pH 7.5, 150 mM NaCl, and 0.1% NP‐40) and transferred to a new tube. After two washes with 1 mL of IP wash buffer, the magnetic beads were resuspended in 40 μL of DEPC H_2_O, and the co‐precipitated phages were lysed at 95°C for 10 min. The concentrations of cattle IgG were determined using BSA as a standard. All samples were tested in triplicate.

### 2.4 Phage PCR and Sequencing

*LSDV* gene fragments were amplified from lysed phages using primers F1 (5′‐TTGTCTTCCTAAGACCGCTTGGCCTCCGACTTGGGGTTAACTAGTTACTCGAGTGCGG‐3′) and R1 (5′‐CCGAACGCAGCAAACTACGC‐3′). These PCR products were used as templates for a second round of PCR with primers F2 (5′‐GAACGACATGGCTACGATCCGACTTTCGTATTCCAGTCAGGTGTGATGCTCGG‐3′) and R2 (5′‐TGTGAGCCAAGGAGTTGxxxxxxxxxxTTGTCTTCCTAAGA CCGCTTGGCCT‐3′), where “xxxxxxxxxx” represents a 10‐nucleotide MGI index sequence for sample identification. PCR products were purified by agarose gel electrophoresis and sequenced on the MGISEQ‐2000 NextSeq platform (BGI) at the National Key Laboratory of Crop Genetic Improvement, Huazhong Agricultural University. Low‐quality sequencing reads were filtered using *fastp* (ver 0.20.0), and PCR amplification‐induced adapter sequences were removed using *cutadapt* (ver 1.18) with default parameters [24]. The resulting clean data were used to identify highly immunogenic proteins as previously described [23]. To identify antigenic peptides specifically recognized by sera from cattle naturally infected with LSDV or vaccinated with GTPV, we utilized the open‐source software tool phip‐stat (https://github.com/lasersonlab/phip‐stat/tree/master) for peptide enrichment analysis. The call‐hits subcommand was used to calculate significantly enriched peptides. According to the annotations in the source code, peptides with a hit value greater than one were selected as enriched peptides for further analysis.

### 2.5 Antigen Design for Differential Diagnosis

From VirScan screening results, we found that highly abundant peptides corresponding to the LSDV103 region (amino acids 1–190) were specifically enriched in sera from LSDV‐infected cattle. Notably, a peptide derived from LSDV103 (amino acids 136–190) was also detected in GTPV‐vaccinated sera and was excluded from further analysis. Consequently, the N‐terminal region amino acids 1–135 of LSDV103 were selected for truncated expression and designated as TrLSDV103. The gene encoding the TrLSDV103 protein was synthesized with *Bam*HI and *Xho*I restriction sites at the 5′ and 3′ ends, respectively, and the codon usage was optimized for prokaryotic expression (Table S1). The synthetic gene was then cloned into a prokaryotic expression system to produce the TrLSDV103 protein.

To confirm the nucleotide sequence variation of *LSDV103* among different LSDV strains, the nucleotide sequences of *LSDV103* from various wild‐type and vaccine strains originating from different countries or regions were downloaded from GenBank and analyzed using BLAST.

### 2.6 Production of Recombinant Antigen

To express the TrLSDV103 protein, *E*. *coli* BL21(DE3) cells (TransGen, Beijing, China) harboring the pET‐28a expression vector were cultured in 500 mL of LB medium supplemented with 50 mg/mL kanamycin at 37°C with continuous shaking. The cultures were grown to the mid‐logarithmic phase, indicated by an OD_600_ value ranging from 0.6 to 0.8. TrLSDV103 protein expression was then induced by adding isopropyl‐β‐D‐thiogalactoside (IPTG) (Biosharp, Beijing, China) at a final concentration of 0.5 mM at 37°C for 5 h. *E. coli* BL21(DE3) cells transformed with the empty pET‐28a (+) vector served as a negative control. The harvested bacterial cells were resuspended in 1 × PBS (0.01 M, pH 7.4), and crushed using a high‐pressure crusher (Life Technologies, Carlsbad, USA). The solution containing TrLSDV103 protein was then centrifuged at 12 000 *g* for 10 min to remove bacterial cell debris, and TrLSDV103 protein in the supernatant was eluted with different concentrations of imidazole using Proteinlso Ni‐NTA Resin His Bind Purification Filler according to the manufacturer's instructions (TransGen, Beijing, China). The eluate was collected, and the purified TrLSDV103 protein solution was concentrated using a 10 kDa ultrafiltration tube (Millipore, Merck, Germany). TrLSDV103 protein was exchanged from high‐concentration imidazole buffer into 1 × PBS by centrifugation at 4°C with 4000 *g*. Finally, the purified protein was analyzed by 12% sodium dodecyl sulfate‐polyacrylamide gel electrophoresis (SDS‐PAGE) and stained with Coomassie Blue R‐250 (Biosharp, Beijing, China). The protein concentration was determined using the Bicinchoninic Acid (BCA) kit (Cowin, Jiangsu, China), and the purified protein was stored at −80°C.

For western blot assay, the purified TrLSDV103 protein was separated by SDS‐PAGE and transferred electrophoretically onto a polyvinylidene difluoride (PVDF) membrane (Millipore, Merck, Germany). The membrane was blocked with 5% (w/v) skim milk in TBST buffer (TBS containing 0.05% (v/v) Tween‐20) for 2 h at 25°C. It was then incubated with commercial monoclonal antibodies (Abbkine, Wuhan, China) targeting the six histidine tags, which were diluted at a dilution ratio of 1:5000 in advance for 12 h at 4°C. After being washed three times with 1 × TBST buffer, the membrane was incubated with commercial horseradish peroxidase (HRP)‐goat anti‐mouse IgG (Abbkine, Wuhan, China) diluted at a dilution ratio of 1:5000 for 1 h at 25°C. After another round of washes, TrLSDV103 protein bands were visualized using ECL chemiluminescence reagents (Bio‐Rad, Richmond, USA) and a chemiluminescence imaging system.

### 2.7 Evaluation of Differential Diagnostic Function of TrLSDV103 Protein

In our previous research (H. Zhou, X. Yuan, X. Yan, Y. Chen, C. Hu, A. Guo), we demonstrated that the rAXA19967.1 protein exhibited strong reactogenicity with sera from LSDV‐infected or GTPV‐vaccinated cattle. Therefore, in this study, the rAXA19967.1 protein was used as a positive control to evaluate the differential diagnostic function of the TrLSDV103 protein. The concentrations of rAXA19967.1 and TrLSDV103 proteins were determined using a BCA protein assay kit. Subsequently, antibody titers in sera from LSDV‐infected and GTPV‐vaccinated cattle were assessed by rAXA19967.1 protein‐based iELISA.

For western blot assay, equivalent amounts of rAXA19967.1 and TrLSDV103 proteins were prepared. PVDF membranes were incubated with sera containing equivalent antibody titers from infected and vaccinated cattle, respectively. The differential diagnosis function of the TrLSDV103 protein was then evaluated by western blot analysis.

### 2.8 Establishment In‐House Indirect ELISA (iELISA)

To establish an in‐house iELISA, 96‐well plates were coated with TrLSDV103 protein, and the optimal coating concentration was determined. The antigen was serially diluted in twofold reductions ranging from 8 to 0.25 μg/mL in a carbonate buffer solution (0.05 M CBS, pH 9.6). Subsequently, 100 μL of TrLSDV103 protein solution at the same concentration was added to every two columns of the ELISA plates, which were incubated at 37°C for 2 h. After incubation, the plates were washed three times with 300 μL/well of 1 × PBS containing 0.1% (v/v) Tween‐20 (PBST), with each wash lasting 3 min. The plates were then blocked with 200 μL/well of blocking buffer (PBS containing 1% w/v fish gelatin) at 37°C for 1 h. Next, 100 μL/well of serum from LSDV‐infected and GTPV‐vaccinated cattle was separately added to the plates in each column after serum samples had been serially diluted (1:100, 1:200, 1:400, 1:800, 1:1600, 1:3200, 1:6400, and 1:12800).

Then, TrLSDV103 protein‐coated plates were incubated under four different conditions: 37°C for 1 h, 37°C for 2 h, 4°C for 16 h, and 37°C for 2 h, transferred to 4°C, and stood for 14 h. Additionally, TrLSDV103 protein was blocked at 37°C using various blocking reagents, including 5% (w/v) skim milk, 2% (w/v) bovine serum albumin (BSA), and 1% (w/v) fish gelatin. Furthermore, the blocking solution was applied for different durations (1, 1.5, 2, 2.5, and 3 h) at 37°C, and commercial rabbit anti‐bovine IgG/HRP (Solarbio, Beijing, China) was diluted at 1:4000, 1:6000, 1:8000, and 1:10000.

Finally, the chromogenic reaction was performed at 37°C for 5, 10, 15, and 20 min using the same method described above to determine the optimal chromogenic reaction time for TrLSDV103‐based iELISA.

The plates coated with TrLSDV103 protein were incubated with serum samples at 37°C for 1 h, followed by three washes with 1 × PBST. Then, 100 μL of rabbit anti‐bovine IgG/HRP, diluted 1:8000 with 1 × PBS, was added to each well and incubated for 1 h at 37°C. After another three washes with 1 × PBST, finally, 100 μL of the substrate 3,3′,5,5′‐tetramethylbenzidine dihydrochloride (TMB, Solarbio, Beijing, China)/H_2_O_2_ substrate was added to each well and incubated at 37°C for 10 min to allow the chromogenic reaction. The reaction was then terminated by adding 50 μL/well of ELISA stop solution (Solarbio, Beijing, China). OD_450_ values were measured using a microplate reader (BMG LABTECH, Offenburg, Germany).

Thirty cattle serum samples at 60 days post‐vaccination with GTPV were detected by in‐house iELISA. The mean and standard deviation (SD) of the cutoff value were calculated, and the cutoff value was determined from the receiver operating characteristic (ROC) curve.

For evaluating the analytical specificity of TrLSDV103‐based iELISA, sera positive for LSDV, BVDV, IBRV, *M*. *tb, P. multocida*, and *M. bovis* were tested. For the analytical sensitivity analysis, both infected and vaccinated sera were serially diluted (1:100 to 1:12800), and OD_450_ values were measured.

### 2.9 Application of In‐House iELISA

In this study, TrLSDV103 based iELISA was applied to detect antibodies in 210 clinical bovine serum samples collected from cattle that were either infected with LSDV, vaccinated with GTPV, or with negative backgrounds. The samples included 70 from LSDV‐infected farms, 120 from GTPV‐vaccinated cattle, and 20 from negative cattle.

### 2.10 Evaluation of the Immunogenicity of TrLSDV103 Protein

To assess the vaccine target potential of TrLSDV103 protein, BALB/c mice were immunized. Five SPF‐grade mice were administered 100 μg of adjuvant‐emulsified TrLSDV103 protein via subcutaneous injection at the neck in two doses, with a 2‐week interval between them. Another five mice received MONTANIDE ISA 201VG (SEPPIC, Paris, France) as a negative control.

Fourteen days after the second immunization, orbital blood was collected from the BALB/c mice. The blood was incubated at 37°C for 30 min, and serum was separated by centrifugation at 3000–4000 *g* for 5 min. IgG antibody levels and titers in the serum were detected using an ELISA IgG kit (MEIMIAN, Jiangsu, China) and an iELISA method based on TrLSDV103 protein.

To evaluate cytokine expression in the mouse serum on days 14 and 28 post‐immunization (dpi), quantitative analysis was performed using commercial ELISA kits. The cytokines detected included interferon‐γ (IFN‐γ), interleukin‐6 (IL‐6), tumor necrosis factor‐α (TNF‐α), and interleukin‐1β (IL‐1β).

Serum from five mice per adjuvant and immunized group was inactivated at 56°C for 30 min, then twofold serially diluted in DMEM (1:5 to 1:640) in triplicates (50 μL/well). GTPV AV41 virus (100 TCID_50_/50 μL) was added to 96‐well plates with diluted sera (50 μL/well). Controls included toxicity, positive, negative, and cell controls. The plates were incubated at 37°C, 5% CO_2_ for 1 h, followed by the addition of 100 μL of Vero cell suspension (2–5 × 10^5^ cells/mL). After gentle agitation, the plates were cultured with daily monitoring for cytopathic effects (CPE).

Spleen cells from the adjuvant and immunized mouse groups were isolated under sterile conditions. These cells were stained with APC anti‐mouse CD3, FITC anti‐mouse CD4, and PE anti‐mouse CD8a antibodies, which bind to specific antigens on the cell surface. After washing, the number of CD3^+^CD4^+^ and CD3^+^CD8^+^ positive cells in both groups was determined using flow cytometry.

### 2.11 Statistical Analysis

Homology analysis of the nucleotide sequences was performed using the MegAlign software. Detection of % CD3^+^CD4^+^ in mice spleen cells was performed using the FlowJo software. Data analysis was performed using GraphPad Prism 8.3.0 (GraphPad Software, San Diego, CA, USA). The comparison of OD_450_ values between serum samples from LSDV‐infected and GTPV‐vaccinated cattle were evaluated using Student's *t*‐test and two‐way ANOVA test. Statistical significance was indicated by: *p < 0.05, **p < 0.01, ***p < 0.001, and ****p < 0.0001.

## 3 Results

### 3.1 VirScan Platform for the Unbiased and Genome‐Wide Screening of LSDV Highly Immunogenic Peptides

**FIGURE 1:**
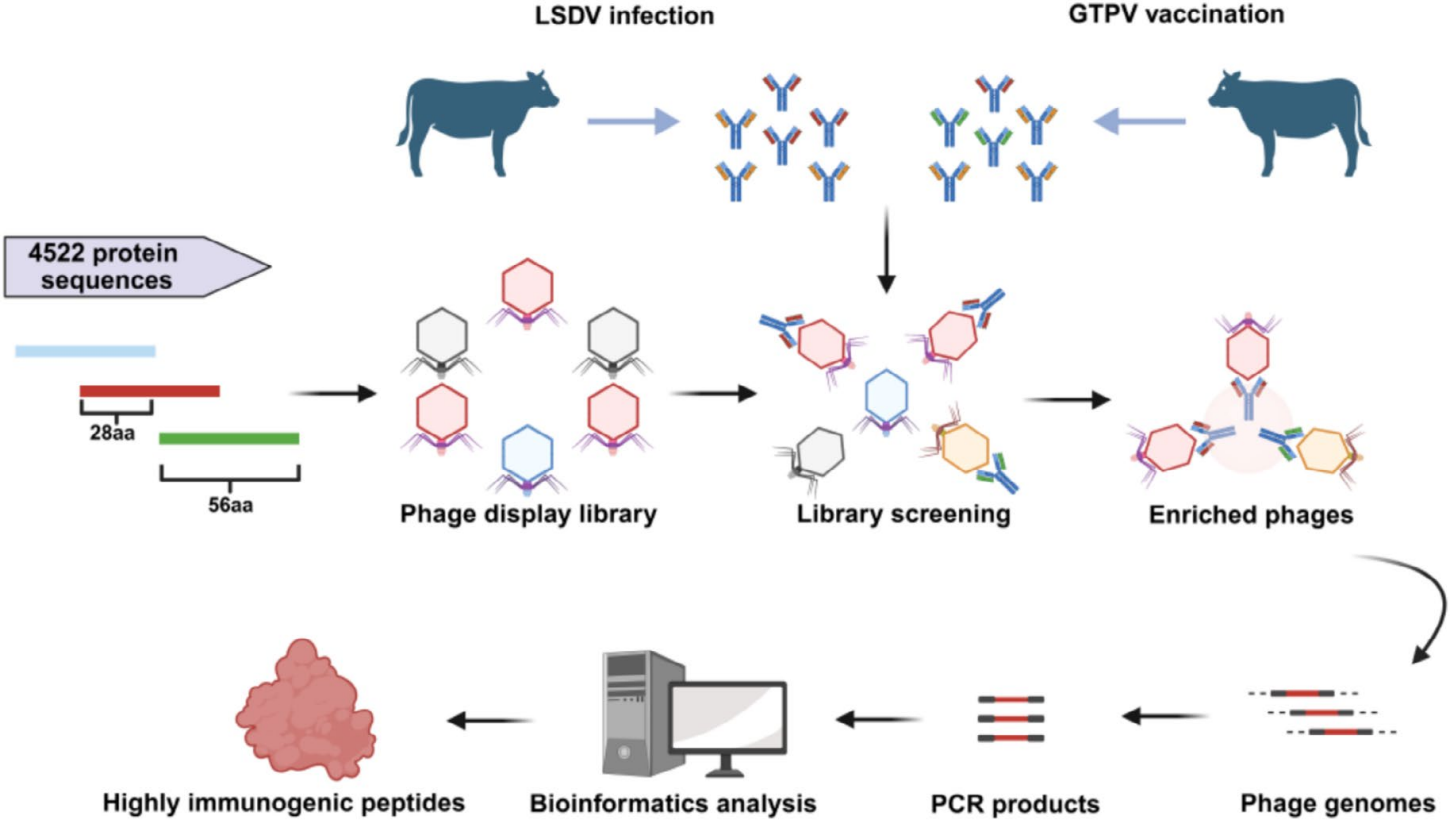
VirScan platform for screening highly immunogenic peptides of LSDV. A phage display library comprising full‐genome sequences from 29 LSDV strains was screened for highly immunogenic peptides using 20 infection and 20 immunized sera. Phages bound to antibodies were recovered using protein A/G beads, amplified by PCR, and sequenced. Bioinformatics analysis revealed that peptides derived from *LSDV103* gene were highly enriched in infection sera. Peptides corresponding to amino acids 136–190, which were also identified in immunized sera, were excluded, leading to the expression of the TrLSDV103 protein.

The phage display library included genomic sequences from 29 LSDV strains, which collectively contained 4522 protein sequences. These sequences were fragmented into 56‐amino acid peptides, and redundancy was removed using a 95% threshold, resulting in a total of 1792 unique peptides. The resulting phage library was utilized to screen for highly immunogenic peptides recognized by sera from LSDV‐infected cattle (Figure 1). Bioinformatics analysis revealed strong enrichment of peptides from LSDV103 (amino acids 1–190) in sera from infected animals. The 136–190 amino acid region of LSDV103 was also recognized by sera from GTPV‐vaccinated cattle. To enable differential serological diagnosis, we deleted this region and expressed the truncated form of LSDV103 (amino acids 1–135, TrLSDV103).

### 3.2 Homology Analysis of TrLSDV103 Protein in Different Strains

Homology analysis of the nucleotide sequences of *TrLSDV103* from 20 LSDV strains was performed using MegAlign software. The *TrLSDV103* gene exhibited high conservation, with nucleotide sequence similarity to other LSDV strains ranging from 99.3% to 100% (Figure S1).

### 3.3 Recombinant Protein Expression

**FIGURE 2:**
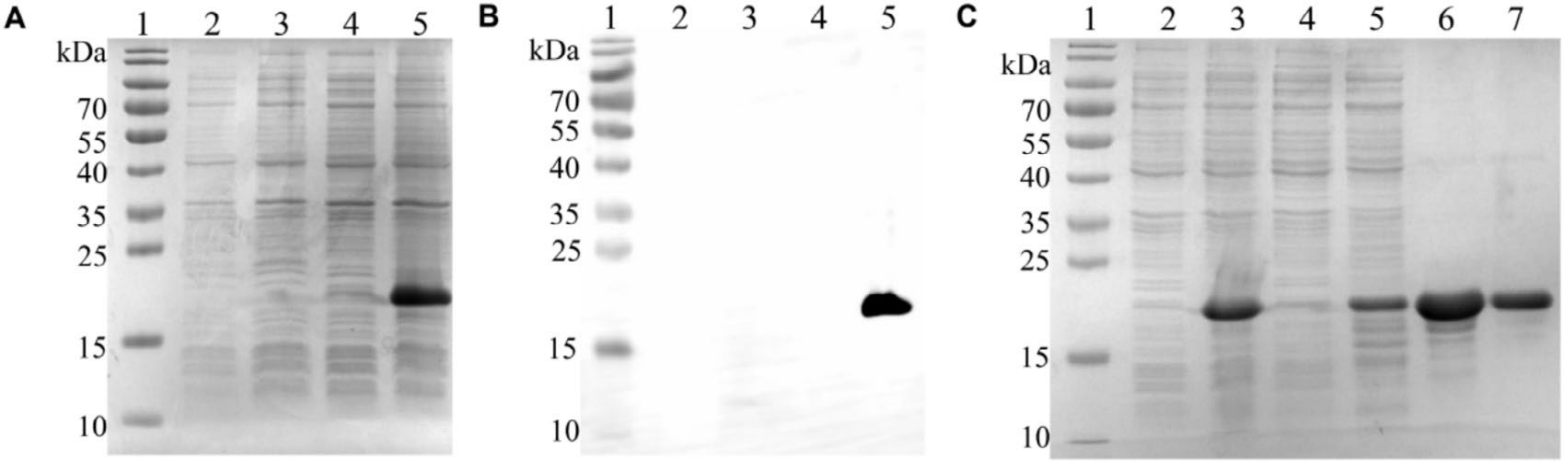
Identification of the expressed and purified TrLSDV103 protein by SDS‐PAGE and western blot assay. (A) SDS‐PAGE analysis of TrLSDV103 protein‐induced expression. (B) Western blot assay of TrLSDV103 protein with the commercial monoclonal antibody to His‐tag. Lane 1, Molecular weight marker; Lane 2, PET‐28a (+); Lane 3, PET‐28a + IPTG; Lane 4, TrLSDV103; Lane 5, TrLSDV103 + IPTG. (C) SDS‐PAGE analysis of the purified TrLSDV103 protein. Lane 1, Molecular weight marker; Lane 2, Pre‐induction TrLSDV103 protein; Lane 3, Post‐induction TrLSDV103 protein (After 5 h); Lane 4, Elution with 10 mM imidazole; Lane 5, Elution with 100 mM imidazole; Lane 6, Elution with 250 mM imidazole; Lane 7, Elution with 500 mM imidazole.

The recombinant plasmid *TrLSDV103* was confirmed by PCR and Sanger sequencing (data not shown). Subsequently, TrLSDV103 protein was successfully expressed in both supernatant fractions and inclusion bodies following IPTG induction in *E. coli* BL21(DE3), with a molecular weight of ~20 kDa (Figure 2A). The successful expression of TrLSDV103 protein was further confirmed by western blot assay, in which a PVDF membrane was incubated with a monoclonal antibody specifically targeting the six histidine tags (Figure 2B). TrLSDV103 protein in the supernatant was purified using a Histrap column with different imidazole concentrations. The optimal elution was achieved with 250 mM and 500 mM imidazole, as confirmed by SDS‐PAGE analysis (Figure 2C).

### 3.4 Differential Diagnosis of TrLSDV103 Protein by iELISA

To evaluate the differential diagnostic potential of TrLSDV103 protein using iELISA, 2 μg/mL of rAXA19967.1 protein, which exhibited strong reactogenicity with sera from LSDV‐infected or GTPV‐vaccinated cattle, was first used to coat the ELISA plates. The rAXA19967.1‐specific antibody titers in both infected and vaccinated sera were measured. The results showed that the rAXA19967.1‐specific antibody titers were largely consistent between the LSDV‐infected and GTPV‐vaccinated groups (Table S2). However, when the ELISA plates were coated with 2 μg/mL of TrLSDV103 protein, the results indicated that TrLSDV103 showed stronger reactivity with antibodies in LSDV‐infected sera compared with GTPV‐vaccinated sera (Table S3). This suggested that TrLSDV103 protein has differential diagnostic potential when applied in the iELISA detection method.

### 3.5 Differential Diagnosis of TrLSDV103 Protein by Western Blot

**FIGURE 3:**
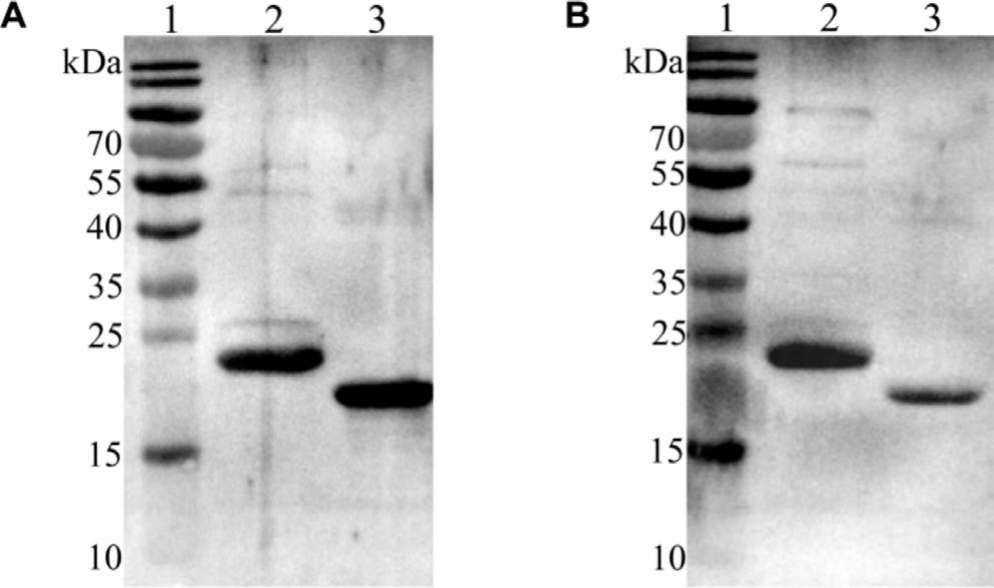
Determination of the differential diagnostic ability of TrLSDV103 protein. (A, B) Western blot assay of TrLSDV103 and rAXA19967.1 proteins using sera from LSDV‐infected and GTPV‐vaccinated cattle, respectively. Lane 1, Molecular weight marker; Lane 2, RAXA19967.1 protein; Lane 3, TrLSDV103 protein.

To demonstrate the differential diagnostic function of TrLSDV103 by western blot, rAXA19967.1 and TrLSDV103 proteins were analyzed by SDS‐PAGE and transferred into PVDF membranes, which were incubated with both infected and vaccinated sera diluted to 1:800. LSDV‐infected serum incubated with TrLSDV103 protein exhibited a clear and specific binding reaction, whereas GTPV‐vaccinated serum incubated with TrLSDV103 protein showed a weak reaction. The rAXA19967.1 protein was used as a positive control. These findings indicated that TrLSDV103 protein could serve as a differential diagnostic target (Figure 3A,B).

### 3.6 Optimization of iELISA Conditions Based on TrLSDV103 Protein

**FIGURE 4:**
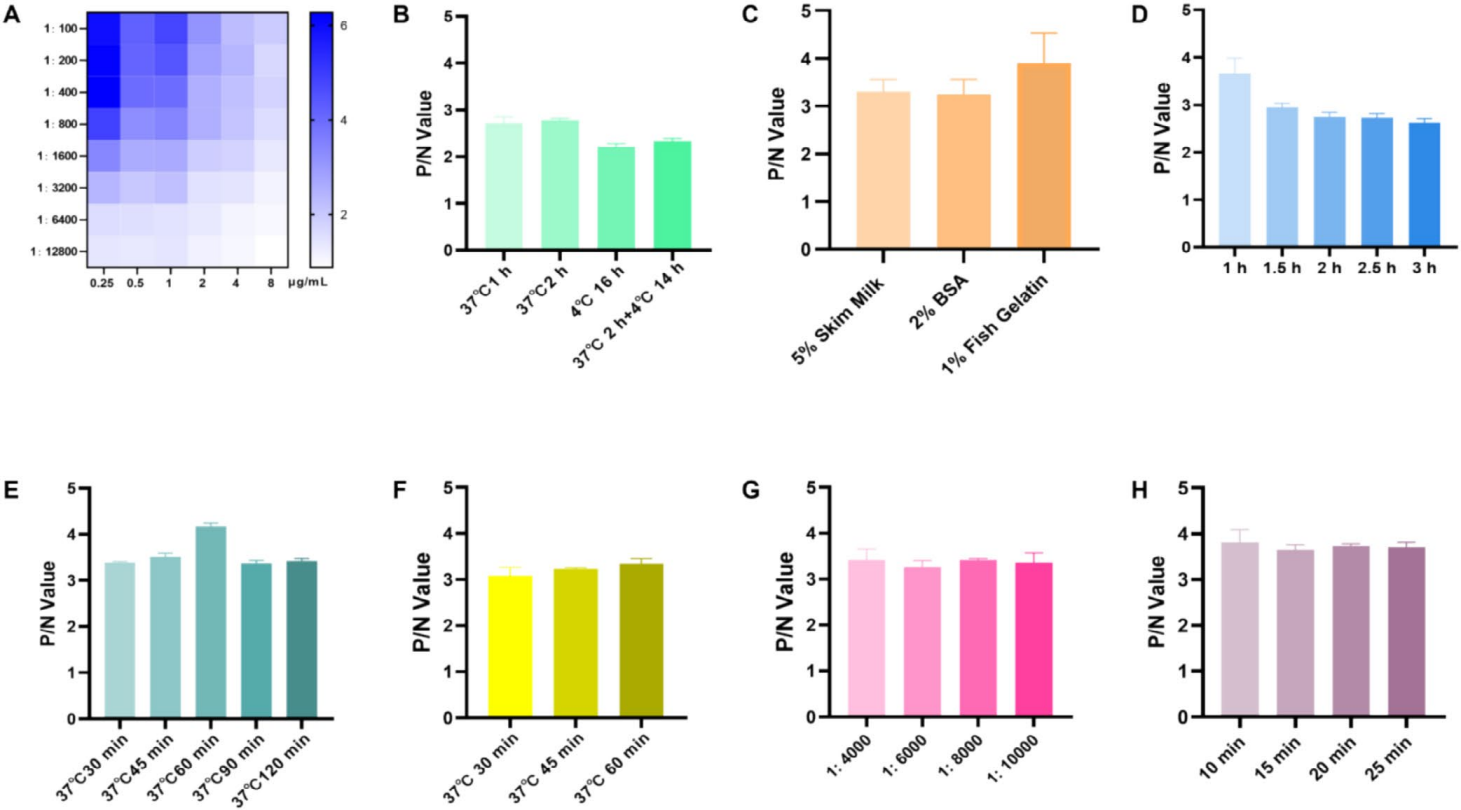
Optimization of reaction conditions for the in‐house iELISA. (A) Optimization of antigen and antisera concentrations. The *X*‐axis represented different antigen concentrations, the left *Y*‐axis showed serum dilutions, and the right *Y*‐axis indicated the P/N ratio, where P referred to the OD_450_ value of infected serum, and N referred to the OD_450_ value of vaccinated serum. (B) Determination of the optimal protein‐coating conditions. (C) Determination of the optimal protein‐blocking reagents. (D) Determination of the optimal blocking time. (E) Determination of the optimal incubation time for serum samples. (F) Determination of the optimal incubation time for commercial rabbit anti‐bovine IgG/HRP. (G) Determination of the optimal dilution of commercial rabbit anti‐bovine IgG/HRP. (H) Determination of the optimal incubation time for TMB/H_2_O_2_ substrate solution. The *Y*‐axis represented the P/N value, indicating the ratio of OD_450_ value between infected serum (P) and vaccinated serum (N) as detected by the in‐house iELISA.

The optimal coating concentration for TrLSDV103 protein was determined to be 1 μg/mL, and the dilution ratio for both positive (P) and negative (N) sera was set at 1:800. The OD_450_ values for positive and negative sera were 1.029 and 0.299, respectively (Figure 4A). P/N values were calculated separately for three replicates of each condition. The data showed that the optimal condition was 37°C for 2 h for coating TrLSDV103 protein (Figure 4B). The optimum blocking condition was determined to be in 1% (w/v) fish gelatin for 1 h at 37°C (Figure 4C,D). The optimal incubation time for serum samples was determined to be 1 h at 37°C (Figure 4E). The appropriate working condition for incubation with rabbit anti‐bovine IgG/HRP was 37°C for 1 h with a dilution of 1:8000 (Figure 4F,G). The highest P/N ratio of OD_450_ value was 3.796 when TMB/H_2_O_2_ interaction time was optimized as 37°C for 10 min (Figure 4H).

### 3.7 Determination of Cutoff Value for iELISA

**FIGURE 5:**
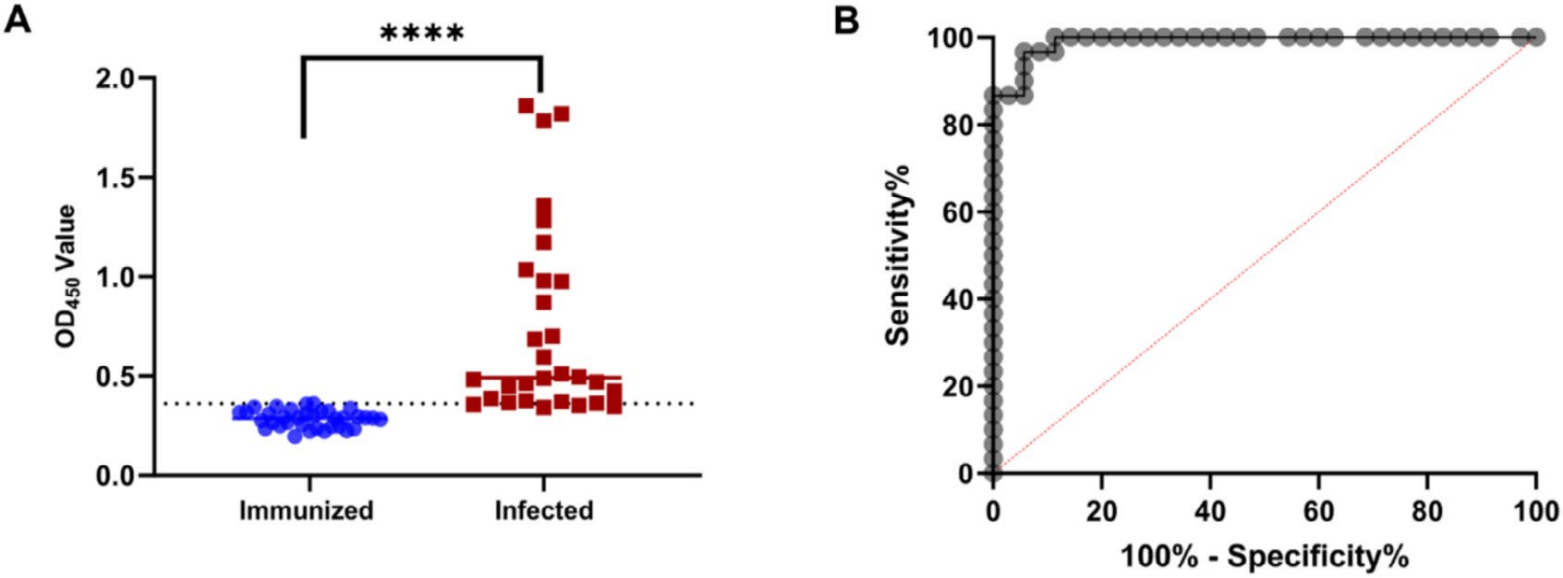
Detection of LSDV‐infected and GTPV‐vaccinated serum samples by the in‐house iELISA. A receiver operating characteristic (ROC) curve was generated to determine the cutoff value for the iELISA. (A) OD_450_ values from 30 naturally LSDV‐infected and 35 GTPV‐vaccinated serum samples with known backgrounds were analyzed. (B) Based on the ROC curve, the optimal cutoff value was determined to be 0.361.

To determine the cutoff value of iELISA, 30 naturally LSDV‐infected and 35 GTPV‐vaccinated serum samples with known backgrounds were tested. We observed that serum antibody levels in LSDV‐infected cattle were significantly higher than those in GTPV‐vaccinated cattle (*p* < 0.0001) (Figure 5A). The OD_450_ values were used to plot a receiver operating characteristic (ROC) curve, and the cutoff value determined from the ROC curve was 0.361 (Figure 5B).

### 3.8 Flow Chart of iELISA Detection Methods

The specific workflow for establishing the iELISA method for the differential diagnosis of LSDV serum antibodies based on the TrLSDV103 protein was carried out as follows: ELISA plates were coated with TrLSDV103 protein at a concentration of 1 μg/mL (100 μL/well) and incubated at 37°C for 2 h. After washing, the plates were blocked with fish gelatin and incubated at 37°C for 1 h. Serum samples, diluted 1:800, were then added to the coated plates and incubated at 37°C for 60 min. Subsequently, the plates were incubated with rabbit anti‐bovine IgG/HRP (diluted 1:8000) at 37°C for another 60 min. Finally, TMB/H_2_O_2_ substrate solution was added for color development and incubated at 37°C in the dark for 10 min (Figure S2).

### 3.9 Determination of Diagnostic Sensitivity and Specificity for iELISA

Thirty naturally infected sera and 35 vaccinated sera of known backgrounds were tested and OD_450_ values were read. The results showed that the sensitivity and specificity of iELISA based on TrLSDV103 protein were 86.67% (95% CI: 70.32–94.69) and 100% (95% CI: 90.11–100), respectively (Table S4).

### 3.10 Analytical Specificity and Sensitivity of iELISA

The iELISA results of different pathogen‐specific sera showed no cross‐reactions with positive sera for BVDV, IBRV, *M*. *tb*, *P. multocida* , and *M. bovis* , whereas specific reactions were observed only with LSDV‐positive sera (Table S5). These results demonstrated the specificity sufficient for clinical diagnostic applications. When the infected serum was diluted to 1:6400, the OD_450_ value was 0.451 (OD_450_ ≥ 0.361 was considered positive). Therefore, the lowest detection limit for positive serum was determined to be 1:6400 (Table S6). This dilution is substantially higher than those typically used in clinical settings, indicating that the current sensitivity of our assay fully meets practical diagnostic requirements.

### 3.11 Application of In‐House iELISA

The in‐house iELISA based on the TrLSDV103 protein was used to detect serum antibodies in 210 clinical samples, including 70 from the farms where sporadic LSDV infection was reported, 120 from GTPV‐vaccinated cattle, and 20 from LSDV‐negative cattle. No positive signals were detected in either the negative group (0/20) or the vaccinated group (0/120). In contrast, 11 out of 70 samples from the LSDV‐infected group tested positive.

### 3.12 Evaluation of the Immunogenicity of TrLSDV103 Protein in Mice

**FIGURE 6:**
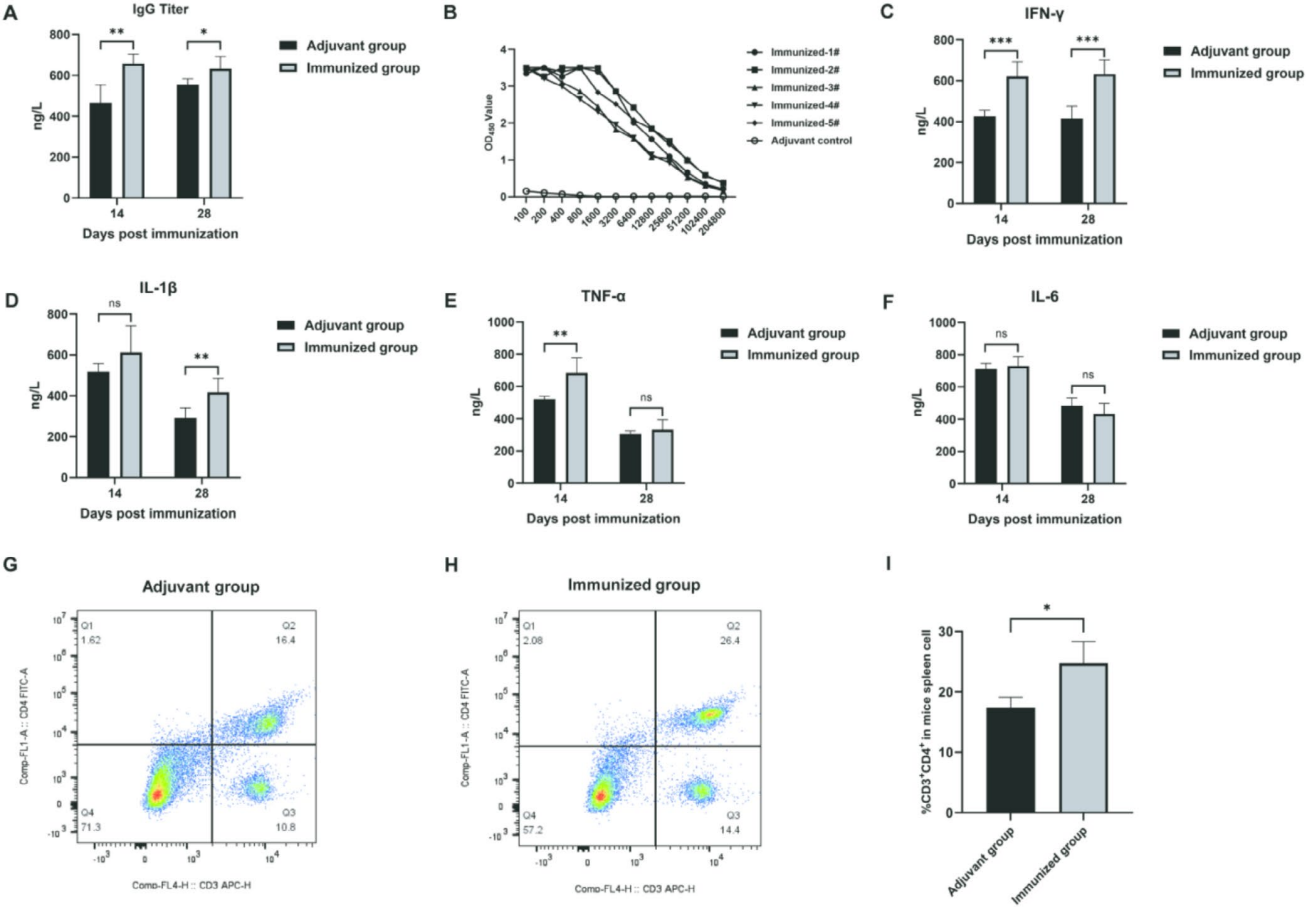
Detection of specific antibodies, cytokine, and %CD3^+^CD4^+^ in BALB/c mice. (A) There was a significant increase in IgG antibody levels in sera from mice immunized with TrLSDV103 protein, compared with the adjuvant group. (B) Serum titers of BALB/c mice were determined using iELISA based TrLSDV103 protein, after the second immunization. Serum levels of the following cytokines were detected in both the adjuvant group and the immunized group at 14 and 28 dpi: (C) IFN‐γ, (D) IL‐1β, (E) TNF‐α, and (F) IL‐6. (G) The proportion of CD3^+^CD4^+^ cell subsets in adjuvant group. (H) The proportion of CD3^+^CD4^+^ cell subsets in immunized group. (I) Comparison of the proportions of CD3^+^CD4^+^ cell subsets between mice immunized with TrLSDV103 protein and those in adjuvant control group *p < 0.05, **p < 0.01, ***p < 0.001, and ****p < 0.0001.

In addition to differential diagnosis, we wanted to assess the potential of TrLSDV103 protein as a vaccine target. To evaluate the immunogenicity of the TrLSDV103, mice were immunized as described in the materials and methods. IgG titers were detected by a commercial IgG ELISA kit, and TrLSDV103‐specific antibody levels were measured using an in‐house iELISA. Sera from mice immunized with the TrLSDV103 protein exhibited significantly elevated IgG antibody levels compared with the adjuvant control group (*p* < 0.05) (Figure 6A). Furthermore, serum antibody levels continued to rise after booster immunization, reaching desirable levels (Figure 6B). These results indicated the strong immunogenic potential of the TrLSDV103 protein. Additionally, the VNT was conducted to assess the neutralizing antibody titers in sera from five BALB/c mice, which ranged from 1:5 to 1:10.

To further investigate the immune response, we measured the levels of cytokines, including IFN‐γ, IL‐1β, TNF‐α, and IL‐6, in the sera of immunized BALB/c mice at 14 and 28 dpi. Compared with the adjuvant group, the concentrations of IFN‐γ and IL‐1β were significantly elevated at 28 dpi in the immunized group (*p* < 0.05) (Figure 6C,D). However, no significant differences were observed for TNF‐α and IL‐6 levels at 28 dpi (Figure 6E,F).

Furthermore, to assess the cellular immune response, spleen cells were aseptically isolated from three mice per group at 14 days after the second immunization. Flow cytometry was used to determine the proportion of CD3^+^CD4^+^ T cell subsets. Mice immunized with TrLSDV103 protein had a significantly higher percentage of CD3^+^CD4^+^ cells compared with the adjuvant group (*p* < 0.05) (Figure 6G–I). These results also suggested that the TrLSDV103 protein elicited a cellular immune response.

## 4 Discussion

This study was the first to employ a novel unbiased genome‐wide approach, called VirScan, for high‐throughput screening to identify highly immunogenic peptides for the development of a DIVA detection method. We investigated the antibody repertoires of both LSDV‐infected and GTPV‐vaccinated cattle. This enabled us to successfully identify both novel and previously known immunogenic peptides of LSDV, offering significant potential for the development of more accurate differential diagnostic tools.

VirScan is a powerful, high‐throughput technique that enables comprehensive profiling of antiviral antibody responses at the epitope level [25]. Its major advantage lies in its ability to screen large peptide libraries across an entire viral proteome, allowing the rapid identification of immunodominant and pathogen‐specific epitopes. However, VirScan relies on short peptides (approximately 56 amino acids in length) displayed on the surface of phages, which limits its ability to detect conformational or discontinuous epitopes that require proper protein folding or post‐translational modifications [22, 26]. As a result, VirScan is biased toward identifying linear epitopes, as highlighted in previous studies [27, 28]. Despite this limitation, the goal of this study was not to identify all possible epitopes of LSDV, but to uncover specific antibody‐binding peptide regions that distinguish natural LSDV infection from GTPV vaccination. Within this context, VirScan provided an effective and efficient solution for identifying differential diagnostic markers.

In this study, we identified a highly immunogenic region within the LSDV103 protein (amino acids 1–135) that was specifically recognized by sera from LSDV‐infected cattle but not by sera from animals vaccinated with the live attenuated GTPV vaccine. This region, termed TrLSDV103, was selected based on differential peptide enrichment in the VirScan analysis and further validated through both iELISA and western blot assays. Importantly, TrLSDV103 is derived from a highly conserved core virion protein, with nucleotide sequence similarity ranging from 99.3% to 100% across diverse LSDV strains, making it a stable and broadly applicable diagnostic target. The recombinant TrLSDV103 protein showed significantly higher reactivity with LSDV‐infected sera compared with GTPV‐vaccinated sera, demonstrating strong potential for use in developing DIVA serological assays. Unlike full‐length protein, which contains overlapping epitopes (amino acids 136–190), the truncated form minimizes non‐specific binding and enhances assay specificity.

Previous studies have explored similar strategies using sheeppox viral antigens, such as ORF095 and ORF103, to distinguish wild‐type infections from vaccine‐induced responses, achieving promising levels of sensitivity and specificity [29]. Furthermore, compared with the existing DIVA target rLSDV‐gap protein [20] against LSDV, the TrLSDV103 protein exhibits two major advantages. Firstly, TrLSDV103 is a naturally occurring protein encoded by the LSDV genome, rather than an artificially designed construct, which preserves its native antigenic characteristics. Secondly, immunization with TrLSDV103 in BALB/c mice induced strong humoral and cellular immune responses, including the generation of detectable neutralizing antibodies. These results highlighted TrLSDV103 as not only a promising differential diagnostic antigen but also a potential vaccine candidate against LSDV. The TrLSDV103‐based iELISA developed in this study demonstrated excellent diagnostic performance, with a specificity of 100% (95% CI: 90.11–100) and a sensitivity of 86.67% (95% CI: 70.32–94.69), supporting its utility in field diagnostics. Given its prokaryotic expression compatibility, sequence conservation, and strong immunoreactivity, TrLSDV103 represents a practical and scalable solution for the serological differentiation of LSDV‐infected from GTPV‐vaccinated cattle, offering critical support for LSD surveillance, control, and eradication programs.

WOAH recommends the VNT as the gold standard for LSDV serology [4]. However, the VNT is complex and time‐consuming, making it unsuitable for large‐scale serum antibody titer screening. ELISA, a routine immunological diagnostic tool, is favored for its simplicity and rapid turnaround time, and has been widely applied in the development of various LSDV detection kits [30, 31]. To be specific, previous researchers have reported a high concordance between commercial ELISA kits and VNT, confirming ELISA as a reliable diagnostic approach [30, 31, 32, 33]. Nevertheless, a critical challenge is to differentiate LSDV‐infected animals from GTPV‐vaccinated ones, which is essential for effective disease control and eradication.

The iELISA based on TrLSDV103 protein yielded a significantly higher OD_450_ value for LSDV‐infected sera than that for GTPV‐vaccinated sera, supporting the potential of TrLSDV103 protein as a differential diagnostic antigen. In this study, a total of 30 naturally LSDV‐infected sera and 35 GTPV‐vaccinated sera were analyzed using TrLSDV103‐based iELISA. Due to the limited number of serum samples with confirmed background information, the diagnostic sensitivity and specificity obtained in this study may have certain limitations. In future studies, LSDV‐infected sera from cattle at different time points should be collected, and the sample size of the serum panel should be expanded to thoroughly validate the diagnostic sensitivity and specificity of the in‐house iELISA, to confirm its capability for early differential diagnosis.

Currently, vaccines for controlling LSD in cattle are primarily categorized into live attenuated and inactivated types. Live attenuated vaccines can be either homologous, for example, the LSDV Neethling strain, which elicits strong immune responses but may pose risks, such as viral recombination and adverse effects [34, 35], or heterologous, for instance, the sheeppox vaccine, which is generally safer but may offer reduced protective efficacy in cattle [36]. Inactivated vaccines are considered safer, but often require multiple doses to achieve and maintain protective immunity [37]. In recent years, novel vaccine platforms, such as subunit and mRNA vaccines, have emerged. These utilize immunoinformatic approaches to identify immunogenic LSDV proteins and design rational vaccine candidates to further evaluate their efficacy [38, 39, 40].

In this study, we used mice, which are widely employed for the preliminary immune evaluation of vaccines and candidate antigens [41, 42], to evaluate the potential of TrLSDV103 as a vaccine target. The immunized BALB/c mice showed significantly elevated IgG antibody levels, confirming the immunogenicity of the TrLSDV103 protein. Neutralizing antibody assays revealed that sera from immunized mice exhibited limited but detectable neutralizing activity. Additionally, levels of IFN‐γ and IL‐1β were significantly increased, suggesting activation of cytokine‐mediated immune responses. Flow cytometry analysis showed a significant increase in CD3^+^CD4^+^ T cell subsets, with no significant change in CD3^+^CD8^+^ cells, indicating activation of helper T‐cell‐mediated immunity. These results indicated the high potential of TrLSDV103 as a vaccine target. However, despite the promising results obtained in mice, further validation studies in cattle are essential to conclusively assess the vaccine potential of TrLSDV103.

In conclusion, the VirScan platform enabled the successful identification of the TrLSDV103 protein as a highly immunogenic and conserved antigen. The TrLSDV103‐based iELISA showed strong potential for differentiating LSDV‐infected animals from GTPV‐vaccinated ones. Furthermore, immunization studies in mice demonstrated that the TrLSDV103 protein could elicit both humoral and cellular immune responses, highlighting its potential as a novel candidate for LSDV vaccine development.

## Author Contributions

**Xinwei Yuan:** methodology, validation, formal analysis, investigation, data curation, writing – original draft. **Junhua Dong:** methodology, software, investigation, validation, writing – original draft. **Zhijie Xiang:** methodology, validation, investigation. **Qian Zhang:** methodology, validation, investigation. **Pan Tao:** formal analysis, writing – review and editing, funding acquisition, project administration, supervision. **Aizhen Guo:** resources, writing – review and editing, funding acquisition, project administration, supervision.

## Ethics Statement

This study was conducted in strict accordance with the recommendations provided in the Guide for the Care and Use of Laboratory Animals of the Ministry of Science and Technology of the People's Republic of China. The Hubei Administrative Committee for Laboratory Animals approved the animal experiments (approval no.:HZAUMO‐2025‐0034).

## Conflicts of Interest

The authors declare no conflicts of interest.

## Supporting information


Figures S1–S2.

Tables S1–S6.

## Data Availability

The data supporting the findings of this study are available in the Figure files and Supporting Information. Additional datasets are available from the corresponding author upon reasonable request.
