# Supplementary material for: A Genome‐Wide Screening of Novel Immunogenic TrLSDV103 Protein of Lumpy Skin Disease Virus and Its Application for DIVA
Source: FASEB J. 2025 May 28;39(11):e70676. doi: 10.1096/fj.202500425R (PMC12117355; doi:10.1096/fj.202500425R)

Figure S1 Sequence homology analysis of TrLSDV103 among different LSDV strains. Nucleotide sequence alignment of TrLSDV103 from 20 LSDV strains using MegAlign software revealed high conservation, with sequence similarity ranging from 99.3% to 100%.


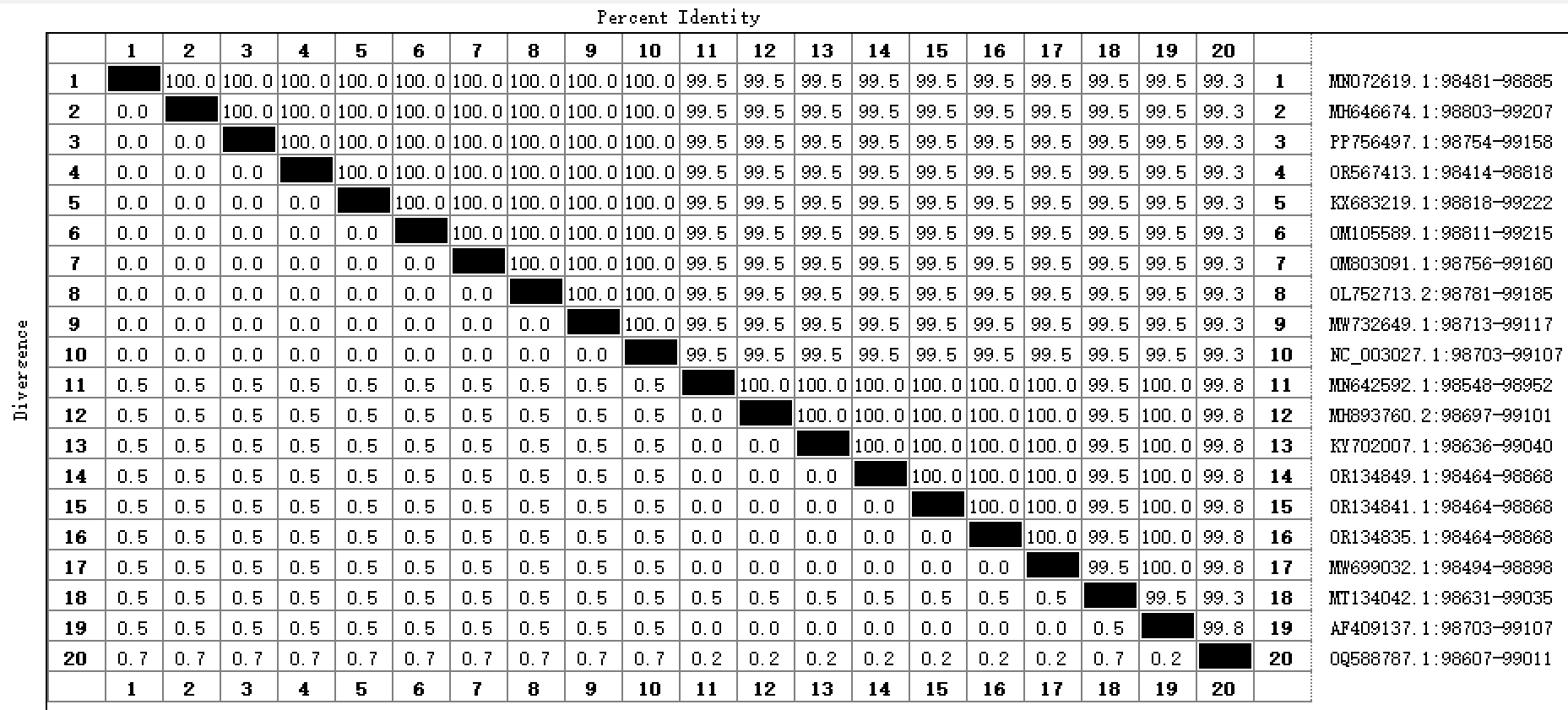


Figure S2 Protocol of iELISA based on the TrLSDV103 protein. The 96-well plates were coated with TrLSDV103 protein at a concentration of 1 μg/mL (100 μL/well) and incubation at 37℃ for 2 h. After washing and blocking, serum samples diluted at 1: 800 were added and incubated at 37℃ for 60 min. Following another wash, the plates were incubated with rabbit anti-bovine IgG/HRP (diluted 1: 8000) at 37℃ for 60 min. Finally, TMB/H_2_O_2_ substrate was added for color development at 37℃ in the dark for 10 min.


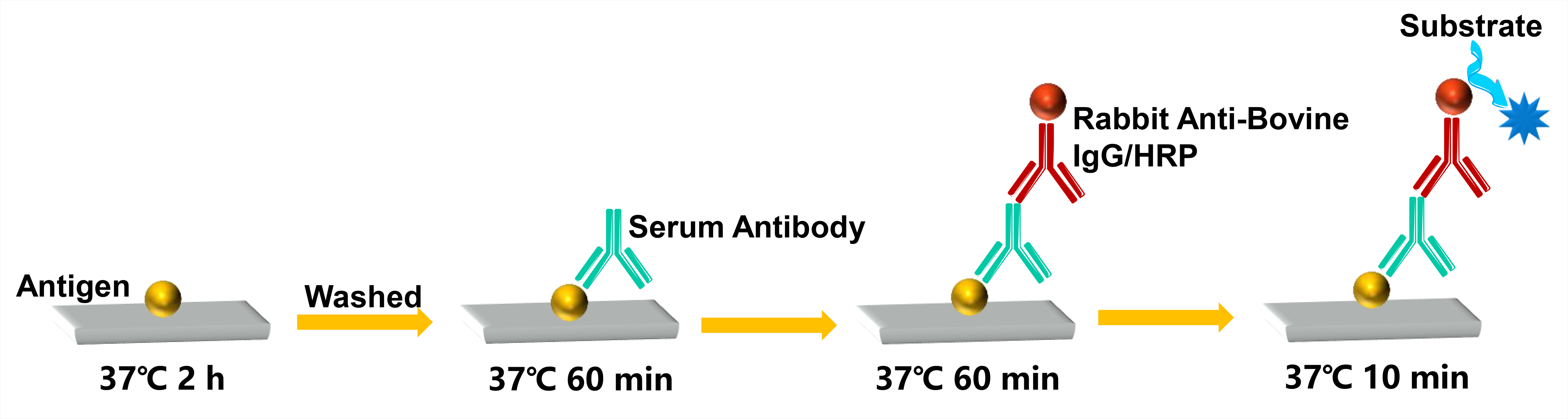

Supplement: Supplementary file 1 — Figures S1–S2. [file FSB2-39-e70676-s001.docx]
