# Supplementary material for: A Genome‐Wide Screening of Novel Immunogenic TrLSDV103 Protein of Lumpy Skin Disease Virus and Its Application for DIVA
Source: FASEB J. 2025 May 28;39(11):e70676. doi: 10.1096/fj.202500425R (PMC12117355; doi:10.1096/fj.202500425R)
Supplement: Supplementary file 2 — Tables S1–S6. [file FSB2-39-e70676-s002.docx]

Table S1 The sequences of TrLSDV103

| The sequences of TrLSDV103 | |
| --- | --- |
| Nucleotide sequence  (5’ to 3’) | GGATCC atg tct gat aaa aaa tta tct cga agc agt tat gat gat tat atc gaa act ata aat aaa cta act cct caa cta agg acc att ctt gcc cac att agt gga gaa caa gca tct caa aaa tca aat ctt act cca gag gat aat act act aat aat aca gat gag aat gaa gta aaa gct ggc aat gtg aaa act aaa gct tgt atg aca aaa cca aat aaa aaa tca aaa tct tgc agt aat aaa caa act act tcg aga agc agt aac gta tgt tct tcg aaa agt gtt aat aat gga gct gtt ttt aaa aaa aga aat aca ttt aac gaa act gat caa ata atg caa gca gta aca aat ggc gga aaa ata gta tac gga acc atg aaa gaa gga aaa tta CTCGAG |
| Amino acid sequence (NH2- to COOH-) | MSDKKLSRSSYDDYIETINKLTPQLRTILAHISGEQASQKSNLTPEDNTTNNTDENEVKAGNVKTKACMTKPNKKSKSCSNKQTTSRSSNVCSSKSVNNGAVFKKRNTFNETDQIMQAVTNGGKIVYGTMKEGKL |

Table S2 Determination of sera antibody titers by the rAXA19967.1 protein based iELISA

| Dilutions | Infected serum OD_450_ | Vaccinated serum OD_450_ |
| --- | --- | --- |
| 1: 100 | 1.172 | 1.142 |
| 1: 200 | 1.048 | 1.043 |
| 1: 400 | 0.827 | 0.904 |
| 1: 800 | 0.710 | 0.803 |
| 1: 1600 | 0.582 | 0.660 |
| 1: 3200 | 0.513 | 0.532 |
| 1: 6400 | 0.445 | 0.428 |
| 1: 12800 | 0.420 | 0.406 |

Table S3 Determination of sera antibody titers by the TrLSDV103 protein based iELISA

| Dilutions | Infected serum OD_450_ | Vaccinated serum OD_450_ |
| --- | --- | --- |
| 1: 100 | 2.741 | 0.815 |
| 1: 200 | 2.234 | 0.732 |
| 1: 400 | 1.761 | 0.582 |
| 1: 800 | 1.321 | 0.507 |
| 1: 1600 | 0.880 | 0.400 |
| 1: 3200 | 0.592 | 0.329 |
| 1: 6400 | 0.401 | 0.268 |
| 1: 12800 | 0.293 | 0.229 |

Table S4 TrLSDV103 protein based iELISA compared with reference sera

|  | | Reference sera | | Total |
| --- | --- | --- | --- | --- |
|  |  | **+** | **−** |  |
| TrLSDV103  based iELISA | **+** | 26 | 0 | 26 |
|  | **−** | 4 | 35 | 39 |
| Total |  | 30 | 35 | 65 |

Table S5 Analytical specificity of iELISA based on the TrLSDV103 protein

| Types of pathogens | LSDV | | BVDV | IBRV | *M*. *tb* | *P*. *multocida* | | *M*. *bovis* | |
| --- | --- | --- | --- | --- | --- | --- | --- | --- | --- |
| OD_450_ values | 1.002 | 0.146 | | 0.287 | 0.346 | | 0.202 | | 0.155 |

Table S6 Analytical sensitivity of iELISA based on the TrLSDV103 protein

| Dilutions | Infected serum OD_450_ | Vaccinated serum OD_450_ | Negative serum OD_450_ |
| --- | --- | --- | --- |
| 1: 100 | 3.107 | 0.812 | 0.195 |
| 1: 200 | 2.685 | 0.613 | 0.188 |
| 1: 400 | 2.191 | 0.492 | 0.183 |
| 1: 800 | 1.560 | 0.423 | 0.183 |
| 1: 1600 | 1.065 | 0.362 | 0.165 |
| 1: 3200 | 0.677 | 0.287 | 0.164 |
| 1: 6400 | 0.451 | 0.245 | 0.153 |
| 1: 12800 | 0.314 | 0.205 | 0.151 |
